# Temperature-Dependent Accommodation of Two Lattices of Largely Different Size during Growth

**DOI:** 10.3390/nano9050710

**Published:** 2019-05-07

**Authors:** Carsten Sprodowski, Karina Morgenstern

**Affiliations:** 1 Institut für Festkörperphysik, Leibniz Universität Hannover, Appelstr. 2, D-30167 Hannover, Germany; carsten.sprodowski@web.de; 2 Lehrstuhl für Physikalische Chemie I, Ruhr-Universität Bochum, Universitätsstr. 150, D-44801 Bochum, Germany

**Keywords:** heteroepitaxial growth, lattice mismatch, scanning tunneling microscopy

## Abstract

If a material grows on another material with a largely different lattice constant, which of the two adapts for an energetically favorable growth? To tackle this question, we investigate the growth of Ag on Cu(111) by variable temperature scanning tunneling microscopy. The structures grown between 120 and 170 K are remarkably different from those grown between 200 and 340 K. The low-temperature structure is rectangular-like and consists of stacked rods, 7 to 8 Ag atoms long, which form a superstructure without long-range order. This structure covers the whole surface prior to nucleation of further layers. The high-temperature structure is hexagonal and consists of misfit dislocations forming 8 × 8 to 10 × 10 superstructures. For this structure, second layer nucleation sets in far before the closure of the first monolayer. While both structures are driven by the large lattice misfit between the two materials, the growing Ag layer adapts to the Cu surface at low temperature, while the Cu surface adapts to the growing Ag layer at higher temperature.

## 1. Introduction

Silver thin films have many potential applications. For instance, they are essential for most optical devices owing to their high reflectance over a wide spectral range. However, the three-dimensional growth of Ag [1,2], caused by a large step edge barrier reducing interlayer mass transport [3,4], limits its usefulness for optical applications. Such a step edge barrier exists in particular for the thermodynamically preferred fcc(111) surfaces.

On the other hand, the Ag/Cu(111) system, at a lattice mismatch between Ag(111) and Cu(111) of 13% with a${}_{\mathrm{Ag}}=0.289$ nm and a${}_{\mathrm{Cu}}=0.255$ nm, has been considered as a representative case of a large adsorbate atom on a substrate with small lattice constant, since its first investigations by Low Energy Electron Diffraction (LEED) [5]. Consequently, it has been a benchmark system for calculations. For Ag/Cu(111), LEED identified a (9 × 9) superstructure with respect to the primitive unit cell of Cu(111) at a coverage of 1 ML deposited at room temperature [5,6]. Scanning tunneling microscopy (STM) data revealed a larger variety of coexisting commensurate superstructures with unit cells of (n,1), n = 8,9,10, with slightly rotated unit cell, after deposition at 373 K at an estimated coverage of 0.3 ML [7]. Within a unit cell, some Ag atoms are vertically modulated in the form of triangles. Two types of coexisting triangle sizes were observed within a mixture of (9 × 9) and (10 × 10) superstructures [8]. The pattern results, as revealed by comparison to theory, from partial dislocation loops in the top-most Cu layer possible through vacancy formation in it [9,10]. The dislocation loops are formed by removal of four or five Cu atoms per triangle facilitating a triangle of stacking fault copper [9,10]. Driving force is the avoidance of on-top positions of Ag atoms, which would be part of a moiré pattern, otherwise expected for two lattices of different lattice constants. For a related system, Au/Ni(111), the formation of either a moiré pattern or a similar dislocation loop pattern depended on temperature [11].

The electronic structure of the system was explored by two-photon photoemission: Room temperature grown Ag islands on Cu(111) exhibit an image state ≈220 meV below the corresponding n = 1 image state of Cu(111) [12]. This state allows monitoring the growth, revealing that only the first two layers grow layer-by-layer. At higher coverage, the Ag/Cu(111) system exhibits Stranski-Krastanov growth leading to quantum well states, whose energy dependence on layer thickness was reproduced theoretically [13,14]. Recent renewed interest into the system results from the plasmonic properties of these structures [15,16,17].

In this article, we explore the structure formation of Ag/Cu(111) by variable temperature scanning tunneling microscopy. The dislocation loop induced network, known from earlier studies of growth at room temperature and above [7], develops at above 200 K. Thus, vacancy formation in the Cu layer, a prerequisite for dislocation network formation, is possible at 200 K and above. Below 170 K, a rectangular superstructure forms. This meta-stable structure has a $\sqrt{3}$ periodicity in Cu〈112〉 directions and exhibits 7 or 8 Ag atoms per 8 or 9 Cu atoms in the 〈110〉 directions. The rectangular symmetry thus differs from the hexagonal one of the support. While the step edge barrier leads to second layer nucleation already at submonolayer coverage for the dislocation network, the absence of island formation for the rectangular superstructure leads to the formation of a complete monolayer prior to second layer nucleation.

## 2. Materials and Methods

STM measurements are performed with a fast-scanning variable-temperature STM of the Aarhus type under ultra-high vacuum (UHV) conditions ($2\times 10^{-10}$ mbar). The Cu(111) sample is prepared by cycles of Ar${}^{+}$-sputtering (1.3 keV, 3 to $5\times 10^{-5}$ mbar, 8 to 15 μA, 10 to 30 min) and annealing up to 970 K (10 to 45 min).

Ag is deposited by resistively heating a short Ag wire attached to a tungsten filament. The surface is held between 120 and 340 K during deposition. The deposition time and rate are varied between 26 and 305 s and between 10${}^{-2}$ and 0.65 ML/min, respectively. During deposition, the chamber pressure stayed below $3\times 10^{-10}$ mbar.

Note that there is a non-linear increase in temperature during deposition on a transfer rod without active cooling. We give the mean temperature of the deposition in the figure captions as calibrated with a K-type thermocouple on the surface of a dummy sample. We stress that the exact temperatures of transition might be sharper in another set-up, but the existence of the two observed structures over tens of Kelvins is not undermined by this temperature rise.

## 3. Results and Discussion

**Figure 1:**
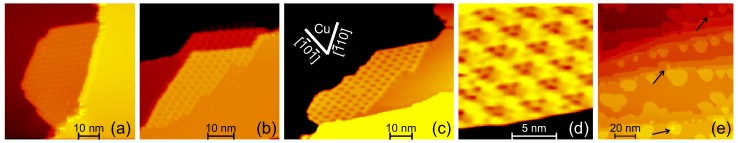
Ag/Cu(111) grown above 200 K: (**a**–**d**) STM images at increasing resolution for room temperature growth; Cu〈110〉 directions as determined from images of atomic resolution are identical for all images (**e**) grown at 230 K, arrows point to second layer nucleation; deposition rates: (**a**,**b**) 0.03 ML/min; (**c**,**d**) 0.11 ML/min; (**e**) 0.2 ML/min; tunneling parameters: (**a**) −1.2 V, 0.56 nA; (**b**) 3.41 V, 4 nA; (**c**) 0.94 V, 0.24 nA; (**d**) 0.94 V, 0.33 nA; (**e**) 1 V, 0.55 nA.

To set the stage, we reproduce the dislocation loop pattern, observed before upon room temperature growth [8] and explore its temperature range. At room temperature, the Ag forms islands at the bottom of the Cu step edge, which have straight steps along the Cu〈110〉 surface directions (Figure 1a–c). The apparent height of the islands with respect to the pristine substrate varies across an island, being a factor of 1.06 to 1.19 larger than the Cu(111) step. This value should be compared to the height of the respective surface steps $h_{\mathrm{Ag}}/h_{\mathrm{Cu}}=0.289\;\mathrm{nm}/0.255\;\mathrm{nm}=1.33$. The islands are thus of monatomic height with a measured height difference of mainly geometric origin. At higher resolution, the surface of the islands exhibits a regular pattern of triangles (Figure 1c) with depressions at the corners (Figure 1d). These triangles result from the dislocation network in the substrate beneath the islands discussed in the introduction. Thus, for these structures, the surface adapts to the a growing Ag(111) layer. The Ag layer keeps its lattice constant and merely relaxes vertically due to the corrugation of the surface layer induced by the misfit dislocations.

As the removal of substrate atoms has a substantial energy barrier, it should be suppressed at lower growth temperature. Indeed, the lowest growth temperature, at which we observe such islands, is approx. 200 K. At such lower growth temperature, the island density is higher (Figure 1e), but their surface exhibits the same pattern. Initially, only the lower side of the step edges are decorated by islands. At higher coverage, islands also nucleate at the upper step edge at the interface between Cu and Ag (Figure 1e). Second layer nucleation is observed already at approx. 0.2 ML coverage, i.e., far below closure of the first monolayer (arrows in Figure 1e).

**Figure 2:**
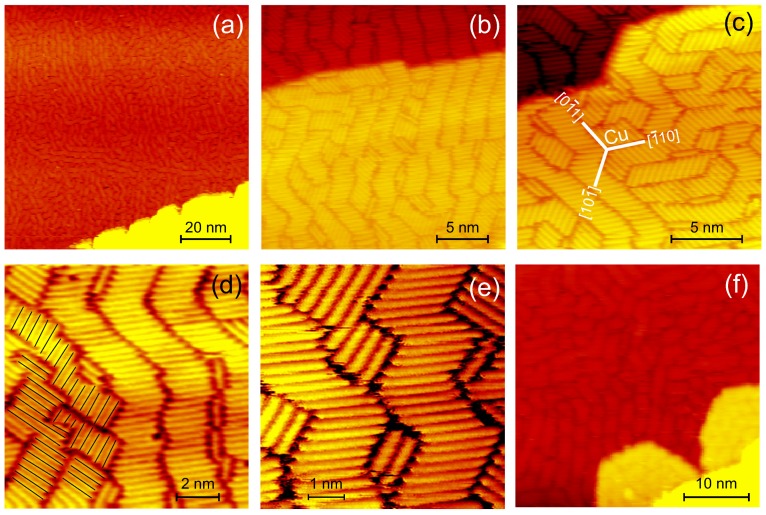
Ag/Cu(111) growth below 170 K: (**a**–**e**) STM images at increasing resolution for approx. 1 ML grown at 112 K (**f**) above 1 ML grown at 135 K; Cu〈110〉 directions were determined from images of pristine surface with atomic resolution; lines in (**d**) symbolize length analysis (see text) deposition rates: (**a**,**f**) 0.65 ML/min; (**c**,**e**) 0.5 ML/min; tunneling parameters: (**a**) 2.1 V, 104 K; (**b**) 0.40 nA, 1.06 V; (**c**) 0.39 nA, 1.06 V; (**d**) 0.39 nA, 1.06 V; (**e**) 7.15 nA, −1.32 V; (**f**) 0.36 nA, 0.94 V.

The growth behaviour is markedly different at lower temperature between 120 and 170 K. At low coverage, there is no indication of the adsorbates in the STM images. Only by scanning at elevated voltage [18] or after deposition of a full monolayer, the adsorbates get visible. Then, the whole surface is completely covered at uniform height (Figure 2a). This monolayer consists of a pattern of meandering stripes extending over several 10 nm at a width between approx. 1 and 2 nm (Figure 2b). Though the overall orientation of the stripes is in the three equivalent Cu〈110〉 directions, they are wavy around these directions (Figure 2c).

Higher resolution reveals that each stripe consists of parallel rods of similar length (Figure 2d). Note that rods of shorter lengths are mainly observed in regions, where stripes of different orientations exist and meet (Figure 2d, lower part) and between meandering stripes (Figure 2e).

Growth extends from step edges in the second layer only at more than one monolayer coverage (Figure 2f). These second layer islands consists likewise of stacked rods forming stripes. The uniform coverage of the surface by one monolayer before nucleation of the second monolayer is possible as there is no island growth, but the layer nucleates only at full monolayer coverages.

For understanding the driving force for the formation of this unique superstructure, we determine its geometrical characteristics. The rods are equally spaced, separated by (0.36±0.03) nm in the Cu〈112〉 directions corresponding to the next-nearest neighbor distance of Cu(111), often referred to as $\sqrt{3}$ distance. At such a distance, each stripe occupies equivalent sites on the surface.

**Figure 3:**
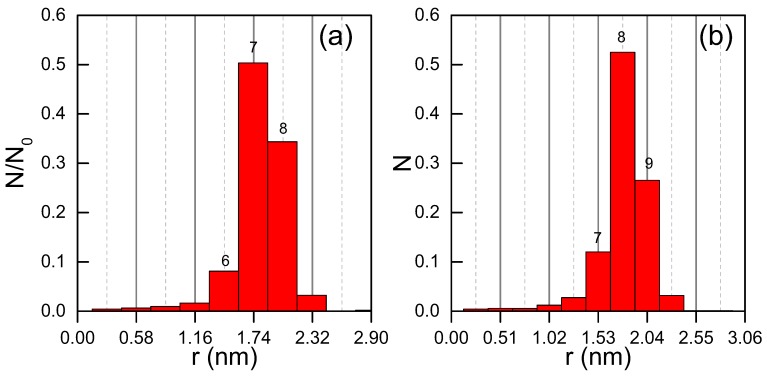
Statistics of rod length at different bin sizes, N${}_{0}$ = 2229: (**a**) $a_{\mathrm{Ag}}=0.289$ nm (**b**) $a_{\mathrm{Cu}}=0.255$ nm; on top of bars corresponds to the number of atoms that form a rod of this length.

For a statistical analysis, we measure the rod lengths for monolayer coverage as indicated in Figure 2d. As the width of the STM tip makes structures look broader than they are, we measure the distance between the points, for which the height rises above approx. 90% of the maximum height of the rod. The vast majority of more than 50% of the rods has a length of 6a${}_{\mathrm{Ag}}$ and another 34% are measured one unit size larger (Figure 3a). In terms of $a_{\mathrm{Cu}}$, 52% have a length of 7 unit cells, 27% are one unit size larger (Figure 3b). A rod of unit length N-1 consists of N atoms. Thus, around 80% of the rods consist of 7 or 8 silver atoms on 8 or 9 Cu sites.

**Figure 4:**
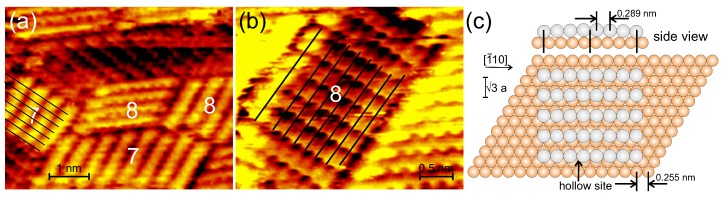
Atomic resolution of rods; number gives length N in atoms: (**a**,**b**) STM images; solid lines mark rows of atoms; deposition rates: 0.65 ML/min; tunneling parameters: (**a**) 1.06 nA, 221 mV; (**b**) 1.07 nA, 186 mV; (**c**) Model: orange balls — Cu atoms, grey balls — silver atoms.

To corroborate our length determination, we attempted to resolve the rods atomically. This turned out to be difficult, presumably because they are only locked in place by the superstructure. Nonetheless, we were successful in a few cases (Figure 4a,b). The majority of the resolved rods has a length of either 7 and 8 atoms as marked in Figure 4. The average distance between the single protrusions corresponds, at ≈0.29 nm, to that between atoms on the Ag(111). The atomically resolved images thus confirm our interpretation of the lengths. We conclude that the Ag keeps its lattice constant and is not or only very slightly compressed. Overall the rectangular superstructure could thus be named $\sqrt{3}x$n with n = 8,9, disregarding the absence of long-range order. Note the similarity between the unit cell length in one dimension and the unit cell size of the two-dimensional superstructure grown at elevated temperature. This suggests that both structures are driven by the lattice mismatch.

A possible explanation for different structures at different temperatures could be a difference in thermal expansion of the two materials. However, the thermal expansion coefficients of Cu and Ag follow the same trend [19,20]. In the region, where we observe the transition from one to the other superstructure, i.e., between 170 and 200 K, the linear thermal expansion is 0.470% for Ag and 0.461% for Cu. A relative change in lattice distance by <10${}^{-4}$ is unlikely the cause for the different structures.

Consequently, we may rationalize the formation of the low temperature structure as follows: As most metals, Ag prefers adsorption in hollow sites. For Ag atoms adjacent in 〈100〉 directions, only the first atom can adsorb in the preferred hollow site. Because of their larger size as compared to the Cu lattice distance, further Ag atoms are increasingly displaced from a hollow site (Figure 4c). The 8th atom is again very close to an fcc site, if the distances between the atoms corresponds to the original Ag(111) distance of 0.289 nm. The lattices match almost perfectly on this larger scale, at a mismatch of 99.2% only for 8 Ag atoms on 9 Cu unit cells (7a${}_{\mathrm{Ag}}/8$a${}_{\mathrm{Cu}}$). One atom more leads to a lattice mismatch of 100.8%; one atom less to 98%. Larger deviations from the best match lead to considerably larger mismatches. The two best matches are indeed those found predominately in our experiments discussed above. In the perpendicular 〈112〉 direction, a similar displacement at closest possible distance would lead to energetically unfavorable on-top sites. These are avoided by the larger distance in these directions. The thus derived atomistic model is drawn in Figure 4c.

We finally discuss, why the rods do not grow larger in length. We discuss three possible reasons. First, the small remaining lattice mismatch could cause a slight displacement from perfect hollow sites. In this case, the reason was a strain effect. Second, two outer atoms in hollow sites stabilize 5 to 6 inner atoms for the rods observed. For a rod of double the length, three atoms in hollow sites would need to stabilize 10 to 12 displaced atoms. In this case, the reason would be thermodynamic. Third, a possible growth process: If the rods were stable entities that diffused over the surface to eventually stack to stripes, then the reason was a kinetic limitation. Observation of longer rods in an induced growth process does not favor this second possible reason [18]. In the same study, the rods appear usually at their full length. We thus tentatively assume that the rod’s length is kinetically limited.

Our analysis suggests that the Ag atoms at the end of the rods, positioned in fcc sites, stabilize the rods. The rods are further stabilized through the stacking of the rods to stripes. The energy gain is not sufficient to stabilize the layer in the form of islands, even at the lowest investigated temperature of 120 K. This, in turn hinders nucleation of the second layer prior to closure of the first monolayer.

For these low temperature structures, the Ag has no structure corresponding to a plane in a Ag crystal. If forms small patches, which are aligned with the Cu substrate directions. While it keeps the preferred closest distance in one direction (horizontal in the model in Figure 4c), it has a much larger distance in the perpendicular direction, a distance, which is given by the substrate. Thus, the growing Ag layer adapts to the the Cu surface at low temperature.

## 4. Conclusions

Our study sheds light on adaption of two lattices with large mismatch under kinetic limitations. The large differences in the lattice spacing between adsorbate and substrate lead to a unique quasi-rectangular superstructure below 170 K. At this temperature the vacancy formation of the thermodynamically preferred dislocation network, developing above 200 K, is hindered. Both superstructures avoid the unfavorable on-top adsorption sites of Ag on Cu, but through different strategies. At low temperature, through large distances between the rods in the adsorbate layer, at high temperature, through dislocations in the substrate layer.

The different formations lead to different growth types: island growth at high temperature and full monolayer condensation at low temperature. While the step edge barrier of the islands leads to the growth of multi-stacked islands at higher temperature, the absence of island formation at lower growth temperature implies a re-entrance of layer-by-layer growth at low temperature. With respect to the formation of flat Ag layers, the layer-by-layer growth at low temperature suggests that on a surface with large lattice mismatch, low temperature growth might be an effective way to overcome the kinetic limitations of the step edge barrier for forming smoother interface layers.

## Abbreviations

The following abbreviations are used in this manuscript:

LEED	Low Energy Electron Diffraction
STM	Scanning Tunneling Microscopy
UHV	Ultra-High Vacuum
DFG	Deutsche Forschungsgemeinschaft
